# Peer effects on sugar-sweetened beverage consumption among children and adolescents: evidence from rural Western China

**DOI:** 10.3389/fnut.2026.1851273

**Published:** 2026-08-05

**Authors:** Chaozhu Li, Zeqi Liu, Xiumei Zhou

**Affiliations:** 1 School of Economics and Management, Huzhou College, Huzhou, China; 2 Public Administration College of Inner Mongolia University, Hohhot, China; 3 Jiyang College, Zhejiang Agriculture and Forestry University, Zhuji, China

**Keywords:** sugar-sweetened beverage, peer effect, rural children and adolescents, nutrition education, generalized propensity scores match

## Abstract

**Introduction:**

Excessive sugar-sweetened beverage (SSB) consumption among children and adolescents in rural areas has become an important public health concern. Although peer influence on adolescent health behaviors has been widely recognized, the mechanisms linking peer SSB consumption to individual intake remain insufficiently understood, particularly in rural settings characterized by boarding education and limited parental supervision. This study examined the association between peer and individual SSB consumption among rural students and explored the mediating role of nutritional cognition.

**Methods:**

Cross-sectional survey data were collected from 1,901 students in 25 primary schools and 11 junior high schools in the Jining District of Ulanqab City, Inner Mongolia, China. Ordinary Least Squares (OLS), Instrumental Variable (IV), and Generalized Propensity Score Matching (GPSM) models were employed to estimate the association and assess the robustness of the findings. Mediation analysis was conducted to examine the role of nutritional cognition, and heterogeneity analyses were performed by boarding status and parental cohabitation.

**Results:**

Peer SSB consumption was positively and significantly associated with individual SSB consumption (*β* = 0.918, *p* < 0.001). Nutritional cognition partially mediated this association. Heterogeneity analyses indicated that the positive association was stronger among boarding students and those not living with their parents than among non-boarding students and those living with their parents.

**Discussion:**

The findings suggest that peer behavior is an important correlate of individual SSB consumption among rural students, partly through its association with nutritional cognition. The stronger association observed among boarding students and those without parental cohabitation highlights the increasing importance of peer influence when parental supervision is limited. These findings support the development of school-based interventions that strengthen positive peer norms and nutrition education to reduce SSB consumption among rural youth, particularly in boarding school settings.

## 1 Introduction

Sugar-sweetened beverage (SSB) consumption has become a staple of daily dietary patterns worldwide, particularly among children and adolescents. Previous studies show that global SSB consumption increased by 16% between 1990 and 2018, with a more pronounced 23% spike among children and adolescents. Compared to high-income and low-income countries, SSB consumption is notably higher in upper-middle-income and lower-middle-income countries (1). Moreover, the most substantial growth in SSB sales has occurred in countries such as China, Chile, Thailand, and Brazil (2).

As the third-largest sugar-consuming country worldwide, China has seen a rapid expansion of its SSB market. Annual beverage production skyrocketed from 9.6 million tons in 1998 to 177.6 million tons in 2019 (3). Concurrently, the proportion of children and adolescents consuming SSBs increased from 73.58% in 2004 to 90.49% in 2011, while average consumption rose from 1.2 L/week to 1.5 L/week (4). Approximately 61.9% of children and adolescents aged 6–17 years consume beverages at least once per week, marking a substantial increase compared with a decade earlier (5).

This explosive growth has sparked severe public health concerns. While some sports nutrition studies suggest that specific sugar-containing beverages can offer functional recovery benefits for athletes under rigorous conditions. For example, Hemmati et al. (6) found that a honey-sweetened beverage could alleviate exercise-induced muscle soreness and facilitate performance recovery among strength-trained females. However, extensive epidemiological evidence proves that long-term, excessive SSB intake drives up the risk of obesity, type 2 diabetes, and cardiovascular diseases (7, 8). A global study estimated that in 2020, SSB consumption was associated with approximately 2.2 million new cases of type 2 diabetes, 1.2 million new cases of cardiovascular disease, and approximately 340,000 deaths across 184 countries (9). In China, deaths attributable to SSB consumption reached roughly 46,000 between 1990 and 2019—a 95% surge from 1990 levels (10). In response, many countries have implemented interventions such as SSB taxes, school sales bans, and health education campaigns (11). However, the efficacy of these measures remains limited, and youth SSB consumption stays stubbornly high. Identifying the core drivers of youth beverage consumption is therefore vital for designing more effective public health policies.

Prior research has demonstrated that SSB consumption among children and adolescents is influenced by multiple factors, including individual cognition, family environment, and the food retail environment (12–14). However, as children and adolescents enter adolescence, their social structures undergo significant changes. The influence of parents and family gradually declines, while the role of peers becomes increasingly prominent (15). As one of the most important social reference groups for young individuals, peers not only shape adolescents’ values and behavioral norms but also influence consumption decisions through mechanisms such as social learning and imitation (16). While peer effects are well-documented in adolescent smoking, alcohol use, and general dietary habits, empirical evidence focusing specifically on SSB consumption among rural youth remains scarce (17–19). However, evidence regarding peer effects on SSB consumption remains relatively limited. A small number of studies have reported a significant positive association between peer beverage consumption and individual beverage consumption (20), but empirical evidence specifically focusing on rural children and adolescents remains scarce. Rural areas in western China provide a unique context for examining this issue. First, compared with urban and rural areas in eastern China, children and adolescents in western rural areas generally exhibit higher levels of SSB consumption (21) and face greater vulnerability in nutritional and health outcomes. Second, these regions have relatively high proportions of left-behind children and widespread boarding school systems, leading to weaker parental supervision and greater reliance on peer interactions in daily life. Therefore, this study focuses on SSB consumption among children and adolescents in rural western China, examining how peer SSB consumption affects individual SSB consumption and exploring the underlying mechanisms.

Compared with existing studies, this study contributes to the literature in three main ways. First, it extends research on peer effects in SSB consumption among rural children and adolescents. Using data from rural western China, this study examines the relationship between peers’ SSB consumption and individual consumption behaviors, providing new empirical evidence on the formation of unhealthy dietary behaviors among rural youth. Second, this study explores potential mechanisms underlying peer effects from the perspective of nutritional cognition and further investigates the heterogeneous effects of family supervision. Building on social cognitive theory, the study incorporates nutritional cognition into its analytical framework to examine whether it mediates the relationship between peers’ SSB consumption and individuals’ SSB consumption. In addition, by considering differences in boarding status and living arrangements (living with parents versus living without parents), this study examines variations in peer effects across different family supervision conditions, providing further insights into the mechanisms underlying the formation of SSB consumption behavior among rural children and adolescents. Third, this study adopts multiple empirical strategies to address potential biases in identifying peer effects. Specifically, to mitigate concerns about shared school environments, peer selection, and omitted variables, this study controls for school fixed effects and employs instrumental variable (IV) estimation and generalized propensity score matching (GPSM) as robustness checks, thereby providing a more cautious assessment of the association between peer SSB consumption and individual consumption behavior.

## 2 Theoretical analysis and research hypotheses

Social cognitive theory posits that behavior is not purely a product of personal preference; rather, it is shaped by continuous interactions among individual traits, environmental factors, and behavior. Within this framework, significant others in the social environment can influence individuals’ behavioral choices through mechanisms such as behavioral modeling, social reinforcement, and feedback. For children and adolescents, peers are among the most important socialization agents beyond the family. As adolescents distance themselves from family-centered networks, they rely heavily on peer groups for validation and emotional support, making their choice patterns highly susceptible to peer dynamics (16).

We expect peer SSB consumption to influence individual youth behaviors through several pathways. First, peer SSB consumption may affect individuals’ decisions through behavioral imitation. Individuals tend to imitate behaviors exhibited by those who share similar characteristics and with whom they have close relationships (22). In school and peer interaction contexts, children and adolescents are frequently exposed to peers’ purchasing and consuming of SSBs. When SSB consumption is prevalent within a peer group, individuals may perceive such behavior as common and acceptable, thereby gradually developing similar consumption habits through imitation. Furthermore, to gain peer acceptance, enhance group belonging, and avoid social exclusion, adolescents often actively adjust their behaviors to align with those of their peers (20).

Second, peer SSB consumption behaviors may influence individual behaviors through social norms. Social norm theory suggests that individuals’ behaviors are strongly influenced by their perceptions of group norms. When SSB consumption is common among peers, individuals may develop descriptive norm perceptions, namely the belief that “most peers engage in this behavior.” Meanwhile, when such behavior is socially accepted or becomes common practice in social interactions, individuals may also develop perceptions of injunctive norms, namely the belief that “this behavior is expected or approved by the group.”

In addition, SSB consumption characteristics further increase the likelihood of peer influence. On the one hand, SSBs are relatively inexpensive, easily accessible, and require minimal effort to consume, making peer imitation more likely. On the other hand, SSB consumption frequently occurs in social contexts such as schools, gatherings, and group activities, making it highly observable and socially interactive. Therefore, compared with less visible health-related behaviors, SSB consumption is more likely to be transmitted and reinforced through everyday peer interactions. Thus, we propose the following hypothesis:

*H1*: Peer SSB consumption is positively associated with individual SSB consumption among children and adolescents.

For children and adolescents, family and peers represent the two most important socialization agents. During development, families shape behavioral preferences through daily interaction, supervision, and value transmission, whereas peers influence behavioral choices through social interaction and group norms. Emotional attachment and normative regulation within the family can effectively restrain behaviors that deviate from healthy behavioral standards. Parents not only restrict children’s exposure to unhealthy behaviors through direct supervision but also enhance children’s self-regulatory abilities through health education and value transmission, thereby reducing the influence of the external environment on individual behavior (32). Previous studies have shown that parental involvement, attention, and dietary rule-setting significantly reduce children’s and adolescents’ consumption of high-sugar, high-fat foods and SSBs (23).

However, in rural China, many children and adolescents experience insufficient parental supervision. On the one hand, labor migration leaves a considerable number of rural children behind while their parents work away from home for extended periods, making consistent daily supervision and behavioral guidance difficult. On the other hand, boarding schools are widespread in rural areas, where students spend substantial time on campus and have limited interaction with parents, thereby weakening family supervision (24). Under such circumstances, children and adolescents increasingly rely on peer groups rather than families for behavioral information and social support. When family emotional support and behavioral guidance are insufficient, adolescents are more likely to seek recognition and support from peers and to imitate peer behaviors to maintain group membership. Existing studies have found that adolescents with weaker parental monitoring are more vulnerable to peer influences regarding smoking, alcohol use, and unhealthy dietary behaviors (25).

Specifically, regarding SSB consumption, boarding students and students not living with their parents often have greater autonomy in their consumption decisions while receiving less immediate dietary supervision and guidance from their families. During school life and peer interactions, they are therefore more likely to be influenced by peer consumption behaviors and group norms. When peers frequently purchase and consume SSBs, these behaviors may be perceived as common and socially acceptable, further influencing individual decisions through imitation and conformity mechanisms. Therefore, compared with adolescents receiving stronger family supervision, those from families with weaker supervision may exhibit stronger peer effects. Accordingly, we propose the following hypotheses:

*H2a*: Compared with non-boarding students, boarding students exhibit a stronger positive association between peer SSB consumption and individual SSB consumption.

*H2b*: Compared with students living with their parents, the positive association between peer SSB consumption and individual SSB consumption is stronger among students not living with their parents.

Peer SSB consumption may not only directly influence individual consumption behaviors but also indirectly affect consumption decisions by altering nutritional awareness. First, peer consumption behaviors may influence individuals’ nutritional cognition. Adolescence is a critical stage of cognitive development. As adolescents become increasingly independent, they gradually reduce their reliance on parental information and pay greater attention to behavioral signals and value judgments conveyed by peer groups (33). When peers frequently consume SSBs and show limited concern for nutrition and health issues, these behaviors may repeatedly convey the message that “SSB consumption is common and acceptable.” With increased exposure, individuals may gradually underestimate the health risks associated with SSB consumption and place less importance on nutrition-related knowledge.

Second, nutritional cognition is an important determinant of dietary behavior. Whether individuals adopt healthy behaviors largely depends on their understanding of health risks and behavioral consequences. Individuals with higher nutritional awareness are generally better able to recognize the potential harms of unhealthy foods and actively avoid unhealthy dietary choices (26). Previous research has demonstrated that nutritional knowledge among children and adolescents is significantly associated with healthy dietary behaviors: individuals with higher nutritional awareness tend to consume more fruits and vegetables and fewer SSBs and other high-sugar foods. Conversely, when individuals lack nutritional knowledge or underestimate the health risks of SSB consumption, their behavioral self-regulation is weakened, making them more likely to develop frequent SSB consumption habits. Therefore, peer SSB consumption may indirectly increase individual SSB consumption by reducing nutritional awareness. Accordingly, we propose the following hypothesis:

*H3*: Peer SSB consumption is negatively associated with nutritional awareness among children and adolescents, thereby indirectly increasing individual SSB consumption.

## 3 Data, variables, and methods

### 3.1 Data sources

The data used in this study were obtained from the “Health and Nutrition Sampling Survey” conducted in Jining District, Ulanqab City, Inner Mongolia Autonomous Region, China, in November 2020. Ulanqab City is located in the agro-pastoral ecotone of northern China, with a relatively high proportion of rural residents, comparatively limited economic development, and a high prevalence of left-behind children and boarding students. Therefore, it provides a representative setting for investigating dietary behaviors and related influencing factors among rural children and adolescents. A stratified cluster sampling method was adopted for sample selection. First, schools in Jining District were stratified by school level (primary and junior high schools) and by location (township and rural areas). Within each stratum, based on schools’ willingness to participate and the feasibility of field investigation, a total of 25 primary schools and 11 junior high schools were selected as sample schools. Subsequently, students in grades five and six of primary schools and grades eight and nine of junior high schools were selected as study participants. If only one class was available in a grade, all students in that class were included; if multiple classes were available, one class was randomly selected for the survey. The survey was conducted through centralized classroom-based questionnaire administration, while was organized by trained investigators with assistance of class teachers. Students completed the Student Basic Information Questionnaire, which collected information on sugar-sweetened beverage consumption behaviors, nutrition knowledge, dietary preferences, and peer relationships. Parents completed the Student Family Background Questionnaire, which collected information on household economic status, parental education level, family guardianship, and parent–child cohabitation status. After completion, investigators conducted on-site checks for questionnaire completeness and logical consistency to ensure data quality. Questionnaires with missing key information or obvious inconsistencies among variables were excluded. Finally, a total of 1,901 valid questionnaires from rural students were included in the analysis.

### 3.2 Variable selection

1. *Dependent variable*: Individual sugar-sweetened beverage (SSB) consumption. According to the Dietary Guidelines for Chinese Residents and related studies, sugar-sweetened beverages (SSBs) are defined as beverages with added sugars (such as sucrose, fructose, glucose, and corn syrup) during production or at the time of consumption. These mainly include carbonated beverages (e.g., cola and Sprite) and sugar-added fruit juice beverages (fruit-flavored drinks with less than 100% fruit juice content). Beverages excluded from this definition include sweetened tea beverages, energy drinks, flavored milk products (e.g., chocolate milk and sugar-sweetened yogurt), plant-based protein beverages (e.g., sweetened soy milk), and 100% pure fruit juice. Individual SSB consumption data were collected via the student questionnaire. Students were asked about the frequency of carbonated beverages and fruit juice consumption during the previous week, as well as the average amount consumed per drinking occasion. The frequency of consumption was categorized into five levels: “0 days,” “1–2 days,” “3–4 days,” “5–6 days,” and “every day.” These categories were assigned corresponding values of 0, 1.5, 3.5, 5.5, and 7 days, respectively.
  The amount consumed was estimated using images of common beverage containers, including 330 mL cans, 500 mL bottles, and 1.25 L bottles, to improve the accuracy of students’ self-reported information. Individual average daily SSB consumption was calculated using the following formula:
  Average daily consumption (mL/day) = (number of drinking days per week × average amount consumed per occasion) ÷ 7.
  The average daily consumption of carbonated and fruit juice beverages was calculated separately for each type, and the two values were then summed to obtain the total average daily SSB consumption (mL/day) for each individual. This indicator simultaneously captures both consumption frequency and consumption volume. Compared with using consumption frequency alone, it provides a more comprehensive assessment of SSB intake among children and adolescents.
2. *Key explanatory variable*: Peer SSB consumption. Building on previous studies on peer effects, this study defines peers as other students in the same class as the individual (20). The classroom is the primary setting where children and adolescents engage in daily learning and social interactions, and it represents an important environment for observing and imitating peer behaviors. Therefore, using the class as the boundary of the peer network is considered appropriate and reasonable. Specifically, peer SSB consumption is defined as the average daily SSB consumption of all other students in the same class, excluding the individual student. It should be noted that the class-level average peer SSB consumption may also be influenced by shared school environments, beverage availability, and health education policies. Therefore, in the subsequent empirical analyses, this study attempts to identify the net effect of peer consumption behavior on individual consumption behavior by controlling for school fixed effects and applying methods including the IV and GPSM (34).
3. *Control variables*: Drawing on previous studies (27, 32), this study selected control variables across three dimensions: individual characteristics, family characteristics, and behavioral preferences. Individual characteristic variables included gender and age. Behavioral preference variables included unhealthy food preferences, which were measured using the food preference scale from the China Health and Nutrition Survey (CHNS). Higher scores indicate a stronger preference for unhealthy foods, such as those high in sugar and fat. Family characteristic variables included whether the child lived with parents, the father’s years of education, the mother’s years of education, whether the child received pocket money, and household income level. These variables can, to some extent, reflect the family socioeconomic environment and individual preference characteristics influencing dietary behaviors among children and adolescents.
4. *Mediator variable*: Nutrition knowledge. The measurement of nutrition knowledge was based on the dietary knowledge scale used in the China Health and Nutrition Survey (CHNS), which consists of 12 items. The items mainly cover aspects such as the relationship between diet and health, the nutritional value of foods, and healthy dietary recommendations. Each item was measured using a 5-point Likert scale, with responses of “strongly disagree,” “disagree,” “neutral,” “agree,” and “strongly agree” assigned scores from 1 to 5, respectively. For negatively worded items, reverse coding was performed before statistical analysis. The scores of the 12 items were summed to generate the total nutrition knowledge score, ranging from 12 to 60 points. Higher scores indicate higher levels of nutrition knowledge. To assess the internal consistency of the scale, a reliability test was conducted, and the Cronbach’s *α* coefficient of the nutrition knowledge scale was 0.86, indicating good reliability.

The meaning of the variables and their descriptive statistics are presented in Table 1.

**Table 1:** Statistical description of variables.

Variable name	Variable description and coding	Mean	Standard deviation
Dependent variable			
SSB consumption	Unit: mL/day	184.419	117.316
Explanatory variable			
Peer SSB consumption	Unit: ml/day (average of classmates, excluding oneself)	178.758	19.873
Mediating variable			
Nutritional cognition	Nutritional cognition score	43.643	4.699
Control variables			
Gender	0 = Female; 1 = Male	0.483	0.500
Age	Unit: years	12.605	1.555
Unhealthy food preference^a^	Unhealthy food preference score	13.933	3.100
Living with parents	0 = No; 1 = Yes	0.811	0.391
Boarding student	0 = No; 1 = Yes	0.301	0.460
Father’s years of education	Actual years of education	9.383	2.701
Mother’s years of education	Actual years of education	8.845	3.100
Pocket money allowance	0 = No; 1 = Yes	0.539	0.500
Household income	Unit: 10,000 yuan/month	0.661	0.630

a This study quantifies unhealthy food preference scores based on the food preference scale from the CHNS, where higher scores indicate a greater inclination toward unhealthy diets, i.e., a stronger preference for junk food consumption. (Scale overview: Please rate how much you like the following foods on a scale of 1 to 5, with 1 representing “dislike very much, “2 representing “dislike, “3 representing “neutral, “4 representing “like,” and 5 representing “like very much.” The items include: 1. u389a2 Fast food (e.g., KFC, pizza, hamburgers, etc.);.2. u390a Salty snacks (e.g., potato chips, crackers, fries, etc.). 3. u391a Fruits; 4. u392a Vegetables; 5. u393a Soft drinks and sugary fruit juices. The scores for u391a and u392a have been reverse-coded accordingly.) Living with parents or one parent is coded as “1 = yes,” while all other situations are coded as “0 = no”.

The average daily SSB consumption for individual rural students in the sample is 184.419 mL, while the mean daily SSB consumption of their classmates (excluding the students themselves) is 178.958 mL. The average nutritional cognition score among sampled rural students is approximately 44, and the average score for unhealthy food preferences is approximately 14. The sample is evenly split between males and females, with ages clustering around 12 years old. Students living with parents and non-boarding students constitute the majority of the rural student sample. Additionally, the average years of schooling for both parents is around 9 years, and the average monthly household income is approximately 6,600 RMB.

### 3.3 Research methods

To identify the association between peer SSB consumption and individual SSB consumption, and to mitigate potential endogeneity to the extent possible, this study employs a series of empirical approaches, including OLS, IV, and GPSM estimation.

First, the OLS model is applied to estimate the baseline association between peer SSB consumption and individual consumption behavior. This model primarily examines the correlation between the two variables. The identification assumption of the OLS estimation is that, after controlling for observable individual characteristics, family characteristics, and school fixed effects, peer SSB consumption behavior is uncorrelated with the model error term. The model is specified as follows:

$$Y_{i}=\alpha_{0}+\alpha_{1}X_{i}+\alpha_{2}Z+\varepsilon_{1}$$ (1)

In Equation 1, $Y_{i}$ represents the individual’s consumption of SSB; $X_{i}$ denotes the mean SSB consumption of other students in the same class (excluding the individual); and *Z* represents the control variables. α_1_ and α_2_ are the coefficients to be estimated, and ε_1_ is the random error term. The price variable is not included in the model primarily because SSB consumption encompasses various beverage types (carbonated beverages and fruit juices), making it difficult to calculate price when analyzing total consumption. To mitigate potential omitted variable bias arising from price factors, this study assumes that students within the same school faced the same SSB prices during the survey period. Based on this assumption, school dummy variables are introduced into the control variables to account for school-level fixed effects (including differences in the price environment).

Given the potential reverse causality between individual and peer consumption, it is necessary to address endogeneity to obtain unbiased parameter estimates. Given the data availability, this study selects peer preference for SSB and peer household income as the instrumental variables combination for peer consumption of SSB. On the one hand, existing studies have shown that consumption preferences and household income are associated with SSB consumption among children and adolescents (28). Therefore, peer preference for SSB and peer household income can influence their SSB consumption, satisfying the relevance criterion for instrumental variables. On the other hand, peer preference for SSB and peer household income reflect peers’ SSB consumption and are not necessarily directly related to the individual’s own consumption, thereby satisfying the exogeneity criterion for instrumental variables.

Mediation effect model. To test Hypothesis 3, this study treats nutrition cognition as a potential mediator variable and, drawing on the mediation effect testing framework proposed by Baron and Kenny (29), constructs the following mediation effect model:

$$Cognize=\gamma_{0}+\gamma_{1}X_{i}+\gamma_{2}Z+\varepsilon_{2}$$ (2)

$$Y_{i}=\beta_{0}+\beta_{1}X_{i}+\beta_{2}Cognize+\beta_{3}Z+\varepsilon_{3}$$ (3)

In the model, cognize denotes nutritional cognition, serving as the mediating variable. The definitions of other variables remain consistent with those in Equation 1.

## 4 Empirical results

### 4.1 Baseline regression results

Table 2 presents the baseline regression estimation results. Model 1 considers only the influence of peers’ SSB consumption on individual SSB consumption among rural students. Models 2 and 3 progressively include control variables and school dummy variables. As shown in the table, compared with Model 1, the R-squared values of Model 2 and Model 3 increase by 0.115 and 0.007, respectively, indicating a continuous improvement in the models’ explanatory power. Moreover, the estimated coefficients for peer SSB consumption are all positive and statistically significant at the 1% level, suggesting that increased peer consumption of SSB strongly explains the rise in students’ individual consumption of SSB; that is, the peer effect exists.

**Table 2:** Baseline regression results.

Variable	Model 1	Model 2	Model 3
Peer SSB consumption	1.035*** (0.133)	0.999*** (0.132)	0.918*** (0.134)
Gender		23.750*** (5.010)	22.081*** (5.013)
Age		−5.595*** (1.844)	6.537* (3.694)
Unhealthy food preference		9.461*** (0.817)	9.547*** (0.815)
Living with parents		−11.501* (6.426)	−9.190 (6.431)
Boarding student		−13.033** (6.176)	−9.363 (6.391)
Father’s years of education		−1.821* (1.072)	−1.676 (1.069)
Mother’s years of education		0.131 (0.945)	−0.037 (0.943)
Pocket money allowance		37.243*** (5.117)	37.739*** (5.103)
Household income		12.848*** (3.868)	13.148*** (3.857)
School fixed effects	No	No	Yes
Constant	−0.553 (23.988)	−167.985*** (48.679)	−288.161*** (58.165)
Observations	1,901	1,901	1,901
*R* ^2^	0.031	0.146	0.153

*, **, and *** denote significance at the 10, 5, and 1% levels, respectively; Standard errors are reported in parentheses; 95% confidence intervals may be computed from standard errors and sample sizes.

From the regression results of Model 3, it can be observed that: First, the estimated coefficient of the core explanatory variable, peers’ SSB consumption, is 0.918 and significant at the 1% level. This indicates that for every 1-unit increase in peers’ SSB consumption, individual rural students’ SSB consumption increases by 0.918 units. Thus, an increase in SSB consumption among peers can significantly increase SSB consumption among individual rural students. The findings support Hypothesis 1. Furthermore, from a public health perspective, even a modest increase in sugar-sweetened beverage consumption may, through long-term accumulation, adversely affect body weight and metabolic health among children and adolescents.

Second, among the control variables, gender has a significant positive impact on rural students’ SSB consumption. That is, compared to girls, boys tend to consume more SSB. Unhealthy food preferences have a significant positive impact on rural students’ SSB consumption, meaning that the stronger their preference for junk food, the more likely they are to consume SSB. The coefficient of having pocket money is significantly positive, indicating that rural students with pocket money consume more SSB than those without. The coefficient on family income is also significantly positive, suggesting that rural students from higher-income families consume more SSB than those from lower-income families.

### 4.2 Heterogeneity analysis

The preceding analysis confirms the promoting effect of peer SSB consumption on individual SSB consumption among rural students. Does this peer effect vary across student groups in different living environments? To answer this, this section examines the heterogeneity of peer effects along two dimensions: boarding status and living with parents.

Table 3 presents the results of the heterogeneity analysis for different groups of rural students. Overall, peer effects significantly promote SSB consumption across all four sub-groups. However, the magnitude of the impact differs. Specifically, for every one-unit increase in peer SSB consumption, consumption increases by 0.929 units for boarding students and 1.242 units for students not living with parents, which are higher than the increases for non-boarding students (0.789 units) and students living with parents (0.871 units).

**Table 3:** Heterogeneity analysis results.

Variable	Boarding students	Non-boarding students	Students living with parents	Students not living with parents
	Model 4	Model 5	Model 6	Model 7
Peer SSB consumption	0.929*** (0.190)	0.789*** (0.211)	0.871*** (0.147)	1.242*** (0.330)
Control variables	Yes	Yes	Yes	Yes
School fixed effects	Yes	Yes	Yes	Yes
Constant	−290.103*** (109.807)	−253.742*** (73.489)	−317.236*** (63.697)	−234.765* (136.857)
Observations	573	1,328	1,542	359
*R* ^2^	0.180	0.137	0.152	0.189

*, **, and *** denote significance at the 10, 5, and 1% levels, respectively; Standard errors are reported in parentheses; 95% confidence intervals may be computed from standard errors and sample sizes.

Compared to non-boarding students and those living with parents, the impact of peer effects on SSB consumption is significantly more pronounced among boarding students and those not living with parents; therefore, the findings support Hypothesis 2. This finding supports the view of Osman et al. (30): adolescents in puberty find it more difficult to establish healthy dietary habits when detached from daily parental supervision and adequate care. Furthermore, an environment characterized by regulatory absence and loose constraints significantly heightens the risk that this group will be influenced by negative peer dietary habits, prompting them to increase SSB consumption by imitating peers’ behaviors.

### 4.3 Endogeneity test

Given potential endogeneity in the baseline regression, this study employs an instrumental variable (IV) approach as a robustness check. The core idea of the IV method is to introduce an exogenous variable that is highly correlated with the endogenous explanatory variable but uncorrelated with the error term, thereby enabling the identification of the causal effect of the endogenous variable. In this study, we select peers’ preference for SSB and peers’ household income as instrumental variables. The rationale is that peers’ dietary preferences and household economic conditions may influence group-level consumption norms, dietary behaviors, and access to SSBs, thereby affecting peers’ SSB consumption levels. Meanwhile, these variables do not directly determine individual students’ SSB consumption behaviors, satisfying the basic requirements for valid instrumental variables.

Table 4 presents the results of the endogeneity test. The first-stage regression results show that the *F*-statistic is statistically significant at the 10% level, suggesting that the selected instrumental variables are relevant. Therefore, OLS estimation may yield biased estimates, and the two-stage least squares (2SLS) method is employed to correct for this bias. Meanwhile, the over-identification test fails to reject the null hypothesis that the instrumental variables are exogenous, and the weak-instrument test indicates that the selected instruments have sufficient explanatory power. These results suggest that the instrumental variable specification adopted in this study is valid (Table 4).

**Table 4:** Endogeneity test results.

Variable	Model 8	Model 9
	First stage	Second stage
Peer preference for SSB	133.491*** (0.000)	
Peer household income	74.938***	
	(0.000)	
Peer SSB consumption		1.111*** (0.000)
Control variables	Yes	Yes
Constant	−832.136*** (0.000)	−330.115*** (0.000)
*R* ^2^	0.283	0.152
Endogeneity test: *F*-statistic	20.91* (0.075)	
Overidentification test	0.584 (0.445)	
Weak instrument test: *F*-statistic	490.655*** (0.000)	

*, **, and *** denote significance at the 10, 5, and 1% levels, respectively; Standard errors are reported in parentheses; 95% confidence intervals may be computed from standard errors and sample sizes.

**Table 5:** Mechanism test results.

Variable	Nutritional cognition	Individual SSB consumption
	Model 10	Model 11
Peer SSB consumption	−0.015*** (0.006)	0.900*** (0.134)
Nutritional cognition		−1.191** (0.536)
Control variables	Yes	Yes
School fixed effects	Yes	Yes
Constant	−253.742*** (73.489)	−290.103*** (109.807)
Observations	1,328	573
*R* ^2^	0.137	0.180

The 2SLS estimation results indicate that, after controlling for endogeneity, peer SSB consumption remains significantly and positively associated with individual students’ SSB consumption among rural students. Specifically, a one-unit increase in peer SSB consumption is associated with an increase of approximately 1.111 units in individual students’ SSB consumption. This finding further supports the robustness of the baseline regression results.

Peers’ preference for SSBs and peers’ household income may partially reflect broader contextual factors, such as shared dietary culture, social norms, or consumption environments at the class or school level. Therefore, the possibility that these instrumental variables affect individual SSB consumption through channels other than peer consumption cannot be ruled out. Accordingly, the IV estimates should be interpreted as approximating the causal relationship under specific identification assumptions, and the related identification limitations are discussed in a later section.

### 4.4 Mechanism analysis

The empirical results above have proven that peer SSB consumption significantly promotes individual SSB consumption among rural students. This section aims to reveal the potential underlying mechanisms. During adolescence, to align their self-image with peers, individuals often conform to the behaviors or opinions of friends and others; meanwhile, this propensity for peer conformity often undermines their independent thinking and mature decision-making. Combined with the previous discussion, we can infer that the cognitive abilities of children and adolescents are highly likely to be influenced by peers, which in turn affects their dietary decisions. Therefore, this paper employs individual nutritional cognition as a reliable mediating variable and uses a mediation effect model to test the validity of this mechanism.

This study employed the Bootstrap method to examine whether peer effects on rural students’ individual SSB consumption operate through a mediating mechanism. Following the mediation analysis model proposed by Hayes and Scharkow (31), a Bootstrap mediation test was conducted. The number of bootstrap samples was set to 1,000. The results showed that, at the 95% confidence interval, the mediation effect was statistically significant, as the confidence interval did not include zero (LLCI [lower limit] = 0.001, ULCI [upper limit] = 0.537). This indicates that the mediating effect in the model is significant. To further illustrate the underlying mediation mechanism, this study further employed a stepwise regression approach for estimation (Table 5).

Model 10 presents the estimation results for Equation 2. It is observed from Model 10 that there is a significant negative correlation between peer SSB consumption and individual nutritional cognition: a 1-unit increase in peer SSB consumption is associated with a 0.015-unit decrease in individual nutritional cognition. This implies that peer SSB consumption negatively affects the nutritional cognition of rural students.

Model 11 presents the regression results for Equation 3. The coefficient for nutritional cognition is significantly negative, while the peer effect remains significantly positive, indicating partial mediation. Combining this with Model 10, we identify two pathways of peer influence: Peer effects directly promote individual SSB consumption among rural students. Peer effects promote SSB consumption by reducing rural students’ individual nutritional cognition. Therefore, the findings support Hypothesis 3.

### 4.5 Robustness checks

Although the baseline regression and IV estimation results indicate a significant positive association between peer SSB consumption and individual SSB consumption, the formation of peer consumption environments is not completely random. Ignoring this issue may lead to biased estimates of peer effects. Therefore, to further reduce potential selection bias arising from observable differences among individuals and to examine the robustness of the findings, this study employed the GPSM method, which is well-suited to continuous treatment variables.

The GPS method requires that the Conditional Independence Assumption (CIA) holds between the treatment variable and the outcome variable:

$$Y\left(t\right)⊥T|X(\forall t\in \left[t_{0},t_{1}\right]$$ (4)

In Equation 4, where T is the treatment variable, representing peer SSB consumption, and Y (t) is the outcome variable, representing the individual rural student’s SSB consumption when the treatment variable takes the value. To ensure the treatment variable lies in [0,1], we remove outliers based on its distribution. We further define the treatment variable as the ratio of peer SSB consumption to the maximum observed in the sample. The vector contains the “matching variables,” also referred to as covariates; these are control variables that influence both the outcome variable and the treatment variable. The covariates selected in this paper include gender, age, unhealthy food preferences, co-residence with parents, boarding status, father’s years of schooling, mother’s years of schooling, household income, and school control variables.

The estimation proceeds in three steps: First, given the covariates X, we estimate the conditional probability density function of the treatment variable T(t). Since the treatment variable lies in the unit interval and exhibits a non-normal distribution, we employ the fractional logit model for estimation. Second, we model the outcome variable Y(t) as a function of the treatment variable T and the Generalized Propensity Score (GPS), and estimate its conditional expectation using OLS. Finally, we estimate the average expected value of the outcome variable Y at treatment level t. We divide the range of the treatment variable into five intervals. For each interval, we estimate the causal effect of peer SSB consumption on individual rural student SSB consumption. By connecting the causal effects across these five intervals, we obtain the “dose–response” function (DRF) relationship between peer SSB consumption and individual consumption over the entire treatment range.

**Figure 1:**
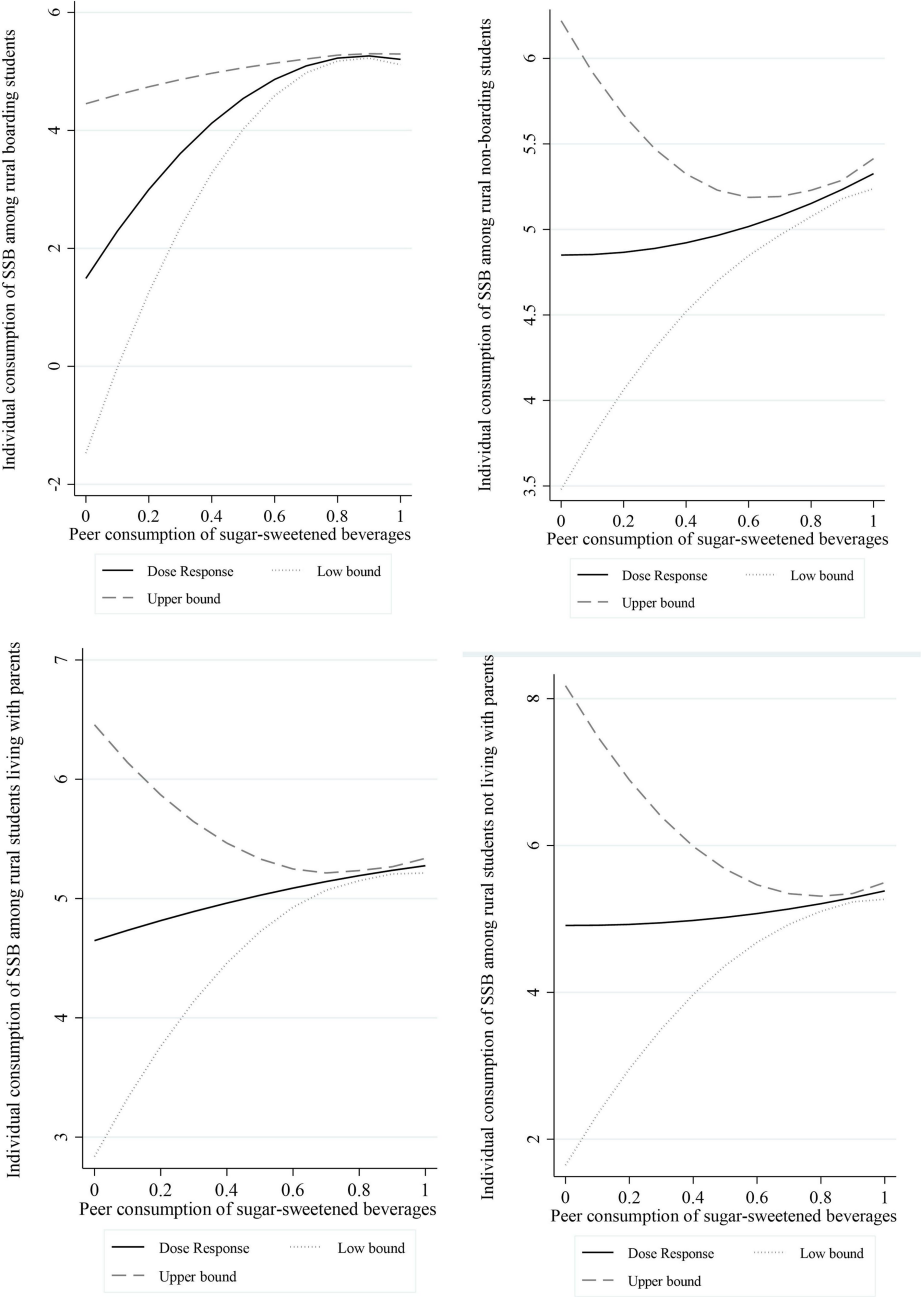
Estimation results of the “dose–response” function of peer effects for different groups.

Figure 1 presents the estimated dose–response functions for different subgroups. The results show that peer SSB consumption appeared to be positively associated with individual SSB consumption among rural non-boarding students living with their parents, with the association following a logarithmic pattern. In contrast, among boarding students and students not living with their parents, peer SSB consumption exhibited a positive exponential association with individual consumption. These patterns were consistent with the direction of findings from the baseline regression and IV analyses, providing further support for the robustness of the results.

Furthermore, among non-boarding students and those living with their parents, the positive association between peer consumption and individual consumption gradually weakened as peer SSB consumption increased. By contrast, among boarding students and those not living with their parents, this positive association grew stronger as peer consumption rose. This difference may reflect variations in social interaction environments and family support across groups. Specifically, boarding students and those not living with their parents generally experience less direct parental supervision and dietary regulation, while spending more time interacting with and living among peers in daily life. Therefore, they may be more susceptible to sustained influences from peers’ dietary behaviors and consumption habits.

## 5 Conclusion and implications

Unhealthy dietary behaviors among rural children and adolescents have received increasing attention, as inappropriate dietary patterns pose significant challenges to their nutritional intake and physical and psychological health. During the critical period when dietary behaviors are established and consolidated, peer groups serve as an important social environment for children and adolescents and may influence individual dietary choices (16). Therefore, a deeper understanding of the mechanisms through which peer effects shape SSB consumption behaviors among children and adolescents is essential for improving nutrition intervention strategies in rural areas.

This study found a significant positive association between peer SSB consumption and individual SSB consumption among children and adolescents, indicating that higher peer SSB consumption was associated with greater individual SSB consumption. This finding is broadly consistent with previous research on peer effects in adolescent dietary behaviors. Existing studies have demonstrated that dietary behaviors among children and adolescents are not solely determined by individual preferences and family environments; rather, peer groups play an important role in shaping food choices, consumption frequency, and health-related behaviors (19). During adolescence, individuals experience an increased need for peer acceptance and a sense of belonging, making them more likely to adjust their behavior through observation, imitation, and social learning. Therefore, peer SSB consumption behaviors may represent an important factor influencing individual beverage choices. The present study extends this evidence by providing additional empirical support among rural children and adolescents.

Furthermore, this study identified significant heterogeneity in the effects of peer consumption. Compared with rural non-boarders living with their parents, peer effects on SSB consumption were stronger among boarding students and those not living with their parents. In addition, the dose–response analysis using GPSM revealed differences in patterns of peer influence across subgroups. Among non-boarders and students living with their parents, peer consumption was positively associated with individual consumption, but this association gradually weakened as peer consumption increased. In contrast, among boarding students and students not living with their parents, the association became increasingly stronger with rising levels of peer consumption. This pattern suggests that when family support and external behavioral constraints are relatively limited, peer consumption behaviors may be more likely to translate into individual consumption practices.

The findings of this study have important public health implications. Given that peer behaviors play an important role in the development of SSB consumption among children and adolescents, schools can serve as key settings for implementing health promotion interventions. First, peer-led health promotion programs could be considered. For example, with guidance from teachers and school administrators, students with a positive influence on peers could be selected and trained as “student health ambassadors.” Through peer role modeling, nutrition education activities, and classroom-based interactive programs, such initiatives may help establish healthier beverage consumption norms among students.

Second, schools could reduce exposure to SSBs by improving the dietary environment. For example, schools may strengthen management of beverage provision in campus stores and cafeterias, restrict the sale of high-sugar beverages, increase access to drinking water and healthier beverage options, and promote healthier choices through environmental strategies such as product placement and pricing adjustments.

Third, more targeted intervention strategies should be developed for specific vulnerable groups. For boarding students and students not living with their parents, who experience greater peer exposure and relatively limited family support, interventions may need to be more intensive and tailored. For example, in boarding schools, residential teachers or trained student leaders could serve as health mentors and organize regular dormitory-based healthy eating activities to encourage positive dietary norms. For students not living with their parents, schools could establish communication mechanisms with actual caregivers, allowing class teachers to provide regular feedback on students’ dietary behaviors and offer practical guidance on healthy eating practices at home.

## 6 Limitations

Although this study systematically examined peer effects on sugar-sweetened beverage (SSB) consumption among rural children and adolescents using multiple econometric approaches, several limitations should be acknowledged.

First, the data used in this study were collected from rural schools in Jining District, Ulanqab City, Inner Mongolia Autonomous Region, western China. Although a stratified cluster sampling method was used, the sample was limited to a single geographic area and may have been influenced by local factors, including socioeconomic conditions, dietary culture, and school management practices. Therefore, caution should be exercised when generalizing the findings to children and adolescents in other regions of China, particularly those with different levels of economic development, or to populations in other countries. Future studies should expand geographic coverage and conduct large-scale, multi-regional investigations to further validate the generalizability of the findings.

Second, SSB consumption data in this study were primarily obtained through student self-reports. Although standardized questionnaires were used and pictures of common beverage containers were provided to help estimate consumption and improve reporting accuracy, recall bias could not be completely eliminated, which may have led to measurement errors. Future studies could integrate multiple sources of information, such as parental reports, school purchase records, and objective consumption records, to improve the accuracy of SSB consumption measurement.

Third, although this study controlled for individual and family characteristics, unhealthy food preferences, and school fixed effects, and further applied instrumental variable (IV) estimation and generalized propensity score matching (GPSM) to reduce potential biases, the influence of unobserved factors cannot be completely ruled out. For example, school beverage supply environments, campus store sales policies, physical activity levels, and family dietary culture may simultaneously affect both peer consumption environments and individual SSB consumption behaviors. Future research should incorporate more comprehensive school- and family-level factors to better understand the mechanisms underlying SSB consumption behaviors.

Fourth, identifying peer effects remains methodologically challenging. In this study, peer SSB consumption was measured as the arithmetic mean of daily SSB consumption among all other students in the same class, excluding the individual student. However, class-level average consumption may also reflect shared environmental factors, such as school dietary policies, beverage availability, and common class-level norms. Therefore, it remains difficult to completely distinguish genuine peer influence from shared environmental effects. Future studies could employ research designs such as random classroom assignment, natural experiments, or longitudinal social network analysis to obtain more accurate estimates of peer effects.

Fifth, the mediation analysis involving nutrition knowledge has certain limitations. Since this study was based on cross-sectional data, the temporal ordering among peer consumption, nutrition knowledge, and individual SSB consumption could not be directly established. Therefore, the mediating role of nutrition knowledge should be interpreted as exploratory evidence regarding potential mechanisms rather than definitive evidence of a causal pathway. In addition to nutrition knowledge, factors such as peer norms, peer pressure, and the need for social belonging may also contribute to how peer behaviors influence individual SSB consumption. Due to data limitations, this study was unable to systematically examine these potential mechanisms. Future research incorporating longitudinal follow-up data and experimental intervention designs is needed to further clarify the dynamic processes and multiple pathways underlying peer effects.

## Data Availability

The raw data supporting the conclusions of this article will be made available by the authors, without undue reservation.
